# The Metabolite Differences in Vascular Smooth Muscle Cells of Abdominal Aortic Aneurysm Revealed by Untargeted Metabolomics

**DOI:** 10.3390/biomedicines14030623

**Published:** 2026-03-11

**Authors:** Yuqi Yi, Ke Hu, Yuxuan Li, Jie Li, Hongping Deng

**Affiliations:** 1 Department of Vascular Surgery, Renmin Hospital of Wuhan University, Wuhan 430060, China; 2024283020136@whu.edu.cn (Y.Y.); rm004312@whu.edu.cn (K.H.); 2 Aortic Abdominal Aneurysm (AAA) Translational Medicine Research Center of Hubei Province, Wuhan 430060, China; 3 Department of Pancreatic Surgery, Union Hospital, Tongji Medical College, Huazhong University of Science and Technology, Wuhan 430022, China; d202482182@hust.edu.cn

**Keywords:** abdominal aortic aneurysm, angiotensin II, metabolomics, vascular smooth muscle cells, farnesoid X receptor, carnitine

## Abstract

**Background**: Abdominal aortic aneurysm (AAA) is a vascular disease with a high mortality rate upon rupture (85–90%). Surgical repair remains the most effective intervention, whereas pharmacological treatments to prevent aneurysm expansion or rupture are limited. Vascular smooth muscle cells (VSMCs) play a crucial role in AAA pathogenesis, and metabolic dysregulation is increasingly recognized as a contributor to disease progression. This study investigated metabolic changes in VSMCs and their association with AAA pathology using untargeted metabolomics. **Methods**: Angiotensin II (Ang II) was used to stimulate rat VSMCs and induce AAA in ApoE−/− mice. Untargeted metabolomic analysis was performed using liquid chromatography–tandem mass spectrometry to detect metabolite changes. Differential metabolites were identified using orthogonal partial least squares discriminant analysis, and metabolic pathways were analyzed using Kyoto Encyclopedia of Genes and Genomes and metabolic set enrichment analysis. **Results**: In Ang II-treated VSMCs, 54 differential metabolites (24 upregulated; 30 downregulated) were identified, whereas 470 differential metabolites (206 upregulated; 264 downregulated) were detected in mouse aortas. Three metabolites—carnitine, lysophosphatidylcholine (0:0/20:4), and 5-hydroxyeicosatetraenoic acid—were common in both models and were enriched in bile secretion and tryptophan metabolism pathways. The carnitine–FXR signaling axis emerged as a potential therapeutic target. **Conclusions**: This study revealed Ang II-induced metabolic changes in VSMCs and their association with AAA pathology. The carnitine–FXR signaling axis may contribute to AAA development, providing new directions for diagnostic biomarkers and therapeutic targets. Future studies should validate these findings in human AAA samples to determine their clinical relevance.

## 1. Introduction

Abdominal aortic aneurysm (AAA) is a vascular disease characterized by segmental, full-layer dilation of the abdominal aorta, defined as an aortic diameter exceeding 50% of the normal value or reaching ≥ 3.0 cm [1]. AAA typically presents without obvious clinical symptoms, but rupture is associated with an extremely high mortality rate of up to 85–90% [1]. Major risk factors for AAA include male sex, advanced age, smoking, atherosclerosis, and a family history of the disease [2]. Currently, open surgery or endovascular repair are the only effective interventions, as no medications are available to prevent aneurysm expansion or rupture [3]. Vascular smooth muscle cells (VSMCs) play a key role in the development and progression of AAA [4,5].

Metabolic disorders refer to the dysregulation of core metabolic pathways, such as carbohydrates, lipids, amino acids, and nucleotides, leading to abnormal concentrations, distribution, and transport rates of intermediate or terminal metabolites that can cause cellular dysfunction and remodeling of organ structures [6,7]. Metabolomics, a technology capable of precisely and efficiently capturing thousands of small-molecule metabolites, can reflect biological information resulting from real-time interactions between the body and environment. This has become an important tool for identifying metabolic disorders in pathological states [8]. In recent years, metabolomics has been increasingly applied to elucidate the pathogenesis of AAA [9,10].

Recent studies have identified metabolic changes within AAA walls, including enhanced glycolysis [11] and the accumulation of lipid peroxidation products [12]. These metabolic changes may drive phenotypic transformation and apoptosis of VSMCs, as well as degradation of the extracellular matrix. However, current research remains limited, and further investigation is required. Given the difficulty of obtaining clinical samples from patients with AAA, this study employed cellular and animal models for experimentation. The classic angiotensin II (Ang II)-infused ApoE−/− mouse model over 28 days is well established and reliable [13,14,15], first reported by Daugherty et al. in 2000 [16]. In the cellular model, stimulating VSMCs with an appropriate concentration of Ang II can effectively replicate key pathological processes observed in AAA [17,18,19]. We hypothesized that the comparative metabolomic profiling of these complementary models would identify potential metabolic alterations and candidate therapeutic targets relevant to AAA pathogenesis. Accordingly, this study integrated Ang II-stimulated rat VSMCs and mouse aortas using untargeted metabolomics to construct a cross-species, multi-scale metabolic continuum. We identified 54 differentially expressed metabolites in VSMCs and 470 in mouse aortas. These metabolites were enriched in multiple pathways, including bile secretion and tryptophan metabolism. By intersecting the results from the two models, we obtained three common metabolites—carnitine, lysophosphatidylcholine (LPC) (0:0/20:4), and 5-hydroxyeicosatetraenoic acid (5-HETE)—and one significantly enriched pathway (bile secretion). We focused on the carnitine–FXR signaling axis as a potential therapeutic target and evaluated its relevance as a peripheral blood biomarker for AAA.

## 2. Materials and Methods

### 2.1. Collection of Animals and Tissues

#### 2.1.1. Cell Culture and Treatment

Preliminary experiments demonstrated that A7R5 cells exhibit stable and reproducible phenotypic changes in response to Ang II stimulation; therefore, this cell line was selected for formal experiments. Cells were purchased from Procell (Wuhan, China; cat. CL-0316, lot 240808010201) as A7R5 VSMCs and cultured in Dulbecco’s Modified Eagle Medium supplemented with 10% fetal bovine serum (Gibco, Waltham, MA, USA) at 37 °C and 5% CO_2_ after resuscitation. Cells between passages 5 and 7 were used for experiments. The experimental group was treated with Ang II (MedChemExpress, Monmouth Junction, NJ, USA), while the control group received an equal volume of phosphate-buffered saline. Each group included three biological replicates (*n* = 3), and experiments were independently repeated three times. Cells were seeded into six-well plates and grown to 60–70% confluence, then stimulated with 1 × 10^−7^ mol/L Ang II for 24 h [20,21,22]. At 80–90% confluence, cells were harvested through trypsinization (Gibco, Waltham, MA, USA) and centrifugation. The resulting pellets were divided for quantitative polymerase chain reaction (qPCR) analysis of angiotensin II type 1 receptor (AT1R), matrix metalloproteinase 2 (MMP2), MMP9, and VCAM expression, while the remaining samples were rapidly frozen in liquid nitrogen and stored at −80 °C for subsequent untargeted metabolomics analysis.

#### 2.1.2. Real-Time Quantitative Polymerase Chain Reaction

Total RNA from VSMCs was extracted using the FastPure^®^ Cell/Tissue Total RNA Isolation Kit V2 (Vazyme, Nanjing, China). The extracted RNA was reverse transcribed and amplified using HiScript III All-in-one RT SuperMix Perfect for qPCR (Vazyme, Nanjing, China). Real-time qPCR was performed using ChamQBlue Universal SYBR qPCR Master Mix (Vazyme, Nanjing, China). Relative gene expression levels were quantified using the average cycle threshold (CT) values and calculated using the 2^−ΔΔCT^ method. Table 1 lists the primer sequences.

#### 2.1.3. ApoE−/− Mouse Abdominal Aortic Aneurysm Model

ApoE−/− mice were obtained from Jiangsu JieCui Yakang Biotechnology Co., Ltd. (Nanjing, China). Male mice aged 8 weeks with body weights of 20–22 g were selected [23]. After inhalation anesthesia with 1.5% isoflurane, a 0.5 cm incision was made in the dorsal scapular region, and the subcutaneous tissue was bluntly separated. An Alzet osmotic pump (model 2004; 28-day release; Alzet, Palo Alto, CA, USA), pre-filled with sterile saline, was loaded with Ang II at a release rate of 1000 ng kg^−1^ min^−1^ [24]. The pump was inserted with its head towards the tail and secured in place, and the incision was sutured. Postoperatively, mice were fed a high-fat diet (XT108C; Jiangsu Xietong Pharmaceutical Biological Engineering Co., Ltd., Nanjing, China) and allowed free access to food and water, with daily monitoring of activity and incision conditions. On day 28, mice were euthanized through anesthetic overdose, the heart–aorta complex was removed intact, and photographs were taken to document aneurysm formation. The suprarenal abdominal aorta was rapidly excised and stored at −80 °C for subsequent untargeted metabolomics analysis. For metabolomic analysis, aortic tissues from three mice were pooled as one biological replicate, with *n* = 3 replicates per group (9 mice per group, 18 mice in total).

#### 2.1.4. Bioinformatics Analysis of Public Transcriptomic Data

To complement the metabolomic findings at the transcriptional level, we analyzed the publicly available dataset GSE261595 from the Gene Expression Omnibus (GEO) database (https://www.ncbi.nlm.nih.gov/geo/query/acc.cgi?acc=GSE261595, accessed on 24 November 2025). This dataset comprises RNA-sequencing data derived from Ang II-infused versus saline-treated ApoE−/− mice. Expression levels of key carnitine regulatory genes, including Bbox1 (gamma-butyrobetaine dioxygenase, involved in carnitine biosynthesis), Cpt1a (carnitine palmitoyltransferase 1A, mitochondrial fatty acid transporter), and Slc22a5 (solute carrier family 22 member 5, organic cation/carnitine transporter), were extracted and compared between experimental and control cohorts.

### 2.2. Untargeted Metabolomics

#### 2.2.1. Sample Preparation

Cell Samples: Cell samples were thawed on ice at −80 °C, and all subsequent procedures were conducted on ice. Methanol–water internal standard extraction solution (500 μL) was added, followed by vortexing for 3 min. Samples were subjected to three freeze–thaw cycles: rapid freezing in liquid nitrogen for 5 min, thawing on dry ice and ice for 5 min each, and vortexed for 2 min. Samples were then centrifuged at 12,000 r/min at 4 °C for 10 min, and 300 μL of supernatant was transferred to a new centrifuge tube and incubated at −20 °C for 30 min. After a second centrifugation at 12,000 r/min at 4 °C for 3 min, 200 μL of supernatant was transferred to the sample vial liners for subsequent analysis.

Tissue Samples: Tissue samples were thawed on ice at −80 °C, and all subsequent procedures were performed on ice. Samples were ground into a fine powder under liquid nitrogen, and 20 mg (±1 mg) was transferred to a centrifuge tube. Methanol–water (70%) internal standard extraction solution (400 μL) was added, followed by vortexing at 1500 r/min for 5 min and incubation on ice for 15 min. Samples were centrifuged at 12,000 r/min at 4 °C for 10 min, and 300 μL of supernatant was transferred to a new tube and incubated at −20 °C for 30 min. After a second centrifugation at 12,000 r/min at 4 °C for 3 min, 200 μL of supernatant was transferred to the sample vial liners for subsequent analysis.

#### 2.2.2. Liquid Chromatography–Tandem Mass Spectrometry Platform

Instrumentation and Chromatography: Metabolomic analysis was performed using a Thermo Vanquish UHPLC system coupled to a Q Exactive HF-X mass spectrometer (Thermo Fisher Scientific, Bremen, Germany). Chromatographic separation was achieved on a Waters ACQUITY Premier HSS T3 column (1.8 µm, 2.1 × 100 mm) (Waters Corporation, Milford, MA, USA) at 40 °C with a flow rate of 0.4 mL min^−1^. Injection volumes were 3 µL for mouse aortic tissue and 4 µL for rat VSMC. The mobile phases consisted of A: 0.1% formic acid in water, and B: 0.1% formic acid in acetonitrile. Supplementary Tables S1 and S2 show the gradient conditions and mass spectrometry parameters.

#### 2.2.3. Raw Data Preprocessing

Raw mass spectrometry files were first converted into mzML format using ProteoWizard, followed by peak extraction and alignment using the XCMS program, with retention time correction. Peaks with a missing rate exceeding 50% across all sample groups were removed, and missing values were imputed with K-nearest neighbor (KNN) imputation plus 1/5 of the minimum value (for values missing >50%, 1/5 of the minimum value was used for imputation; for values missing <50%, KNN imputation was applied). Peak areas were corrected using support vector regression (SVR). Corrected feature peaks were identified by searching laboratory databases and integrating public and predictive databases. Only compounds with an identification score ≥ 0.5 and a coefficient of variation (CV) < 0.3 in quality control (QC) samples were retained. To retain the compounds with the highest qualitative grade and lowest CV, the positive and negative modes were merged to obtain the final all-sample_data.xlsx file.

#### 2.2.4. Metabolite Identification

Metabolite identification was performed by sequentially matching the corrected precise mass (<5 ppm) and secondary fragment spectra as follows: ① in-house standard library (retention time [RT] ± 0.1 min, MS^2^ match > 0.8); ② Human Metabolome Database (HMDB) version 4.0; ③ Kyoto Encyclopedia of Genes and Genomes–Compound (KEGG Compound) database; ④ predictive library (MetFrag). For each spectrum, the highest-scoring match was selected. The comprehensive score was calculated as Score = 0.4 × mass error + 0.6 × spectral similarity. Only identification results with a score ≥ 0.5 were retained. When merging data from positive and negative ionization modes, compounds detected in both modes were represented by the entry with the highest qualitative grade and lowest QC-CV. The final dataset, “all_sample_data.xlsx,” was generated for subsequent statistical and pathway analyses.

#### 2.2.5. Targeted Validation of Carnitine Levels

To independently validate the elevated carnitine levels detected via untargeted metabolomics, free and total carnitine were quantified using the Free Carnitine/Total Carnitine Content Assay Kit (BC0675, Solarbio, Wuhan, China) according to the manufacturer’s instructions. Analyses were performed on both A7R5 VSMCs and mouse aortic tissues to confirm the cross-model consistency of carnitine accumulation observed in the untargeted profiling data.

### 2.3. Data Analysis

Following a conventional untargeted metabolomics workflow, raw data were first converted to the appropriate format. Peak extraction, alignment, and RT correction were performed using XCMS. Ions with a missing rate > 50% were removed, and remaining missing values were imputed using a combination of KNN and 1/5 of the minimum value. Peak areas were corrected via SVR and normalized using unit variance scaling. Only features with QC-CV < 0.3 and identification scores > 0.5 were retained. Principal component analysis (PCA) was initially used to assess sample distribution and remove outliers. Orthogonal partial least squares discriminant analysis (OPLS-DA) was then performed to enhance inter-group differences, which were validated with 200 permutation tests. Variables with a variable importance in projection (VIP) score > 1 were selected. Differential metabolites were defined as those meeting both VIP > 1 and *t*-test *p* < 0.05 criteria and were subsequently annotated using KEGG Compound/Pathway (version 103.0) and HMDB entries (version 5.1), with enrichment significance assessed via hypergeometric testing. Metabolite set enrichment analysis (MSEA, version 6.0) was performed to identify significantly enriched metabolic pathways. Hierarchical clustering and Pearson correlation analyses were conducted on differential metabolites to reveal synergistic or antagonistic relationships following Ang II intervention, visualized through heatmaps, chord diagrams, and network graphs. Finally, intersections of differential metabolites and their associated KEGG pathways were integrated in R (version 4.5.1) for VSMCs and aortic aneurysm tissues, mapping the metabolic continuum between the two.

## 3. Results

### 3.1. Overall Metabolic Changes in VSMCs Induced by Ang II

**Figure 1:**
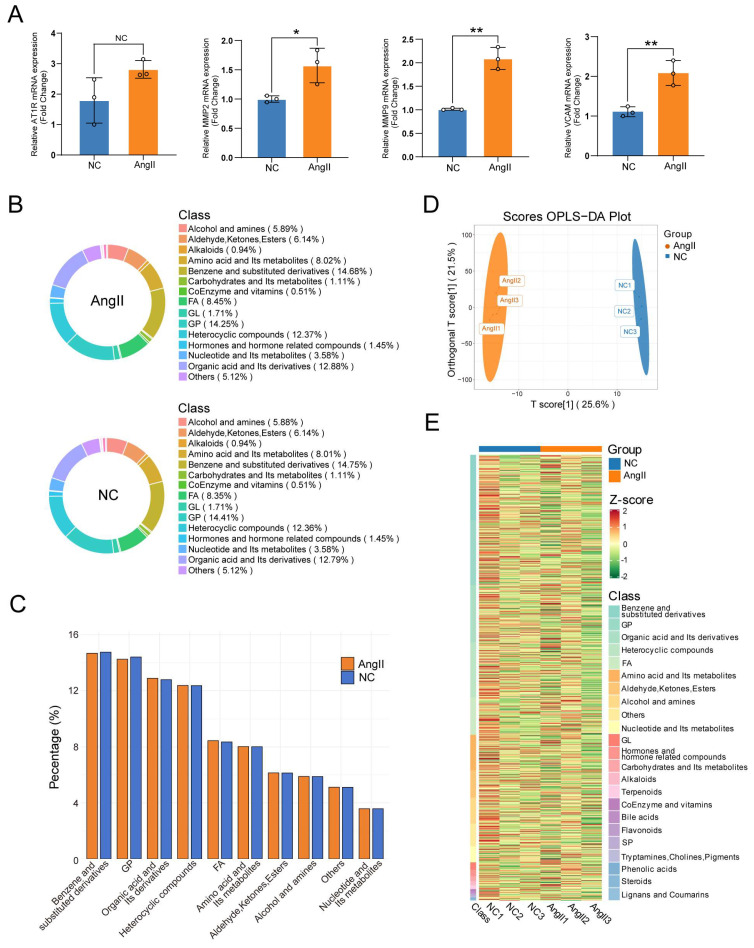
Analysis of the impact of AngII on metabolites in VSMCs. (**A**) mRNA expression bar graphs: mRNA expression changes in related genes after AngII stimulation of rat VSMCs were detected. The data indicate that AngII stimulation leads to a transition of VSMCs from a contractile phenotype to a synthetic phenotype, ** < 0.01, * < 0.05, NC ≥ 0.05; (**B**) metabolite class composition donut charts: each color represents a category of metabolites, and the area of the segments indicates the proportion of each category. Two donut charts are presented, one for the NC group and one for the AngII group. Minor categories with very low abundances are omitted for visual clarity; (**C**) percentage bar graphs: these graphs display the percentages of the top ten metabolite categories in both groups, more clearly distinguishing the differences between groups; (**D**) OPLS-DA score plot: the x-axis represents the predictive principal component (used to distinguish between group differences), and the y-axis represents the orthogonal principal component (used to observe differences within groups). The plot shows a clear separation between the AngII group and the NC group; (**E**) sample overall clustering heatmap: the horizontal axis represents sample names, and the vertical axis represents metabolite information. The “Group” column indicates sample grouping, and the colors represent the standardized processing results of different relative contents (red for high content, green for low content).

Abdominal aortic aneurysm is characterized by VSMC phenotypic switching from a contractile to a synthetic state, accompanied by extracellular matrix degradation and inflammatory activation [25]. Ang II stimulation recapitulates these pathological processes, enabling the establishment of experimental models that mirror key aspects of AAA pathogenesis [26]. To validate the effectiveness of Ang II stimulation in inducing this phenotypic transition, we examined the expression of established molecular markers associated with VSMC dysfunction. Following Ang II stimulation, qPCR analysis revealed the upregulation of AT1R (the primary receptor mediating Ang II effects) [27], MMP2 and MMP9 (key extracellular matrix-degrading enzymes implicated in AAA progression) [28], and VCAM1 (a vascular inflammation marker) [29] (Figure 1A). These findings confirm that our metabolomic analyses were performed on appropriately validated cellular models exhibiting the characteristic phenotypic shift and inflammatory responses observed in AAA [30,31]. Subsequently, untargeted metabolomic profiling was performed, identifying a total of 1026 secondary metabolites across both the experimental and control groups, which served as the basis for subsequent analysis. Table 2 presents the detailed statistics of the identified numbers.

Because metabolite composition is sample-specific, different sample types contain varying categories and proportions of metabolites, and these profiles can also change under different treatments or biological processes. Therefore, the analysis of metabolite composition proportions enables an evaluation of the overall distribution of major metabolites in the samples (Figure 1B). Although the proportions of most metabolite categories were similar between the experimental group (Ang II) and the control group (NC), subtle variations were observed in specific categories, including benzene and its derivatives, and fatty acids. These differences suggest that Ang II treatment may influence specific metabolic pathways, resulting in altered proportions of certain metabolite categories. In addition, organic acids and their derivatives, heterocyclic compounds, and metabolites associated with glycolysis and gluconeogenesis accounted for a large proportion in both groups, demonstrating the ubiquity and biological importance of these metabolite classes and pathways. Data quality and instrument stability were subsequently verified by overlapping total ion current chromatograms from different QC sample mass spectrometry analyses, performing Pearson correlation analysis on QC samples, assessing the stability of internal standards in QC samples, and generating CV distribution plots for all samples (Supplementary Figure S1 and Table S3).

For data analysis, PCA was first applied to obtain an overview of global metabolic differences between groups and the degree of within-group variability (Supplementary Figure S2). To further maximize intergroup differentiation and facilitate the identification of differential metabolites, OPLS-DA was performed, which demonstrated a clear separation between groups with a satisfactory predictive reliability (R^2^Y = 1.000, Q^2^ = 0.613) (Figure 1D and Figure S3). The data were then subjected to unit variance scaling, followed by hierarchical clustering and heatmap visualization using R scripts (Figure 1E).

### 3.2. Screening of Differential Metabolites in VSMCs

**Figure 2:**
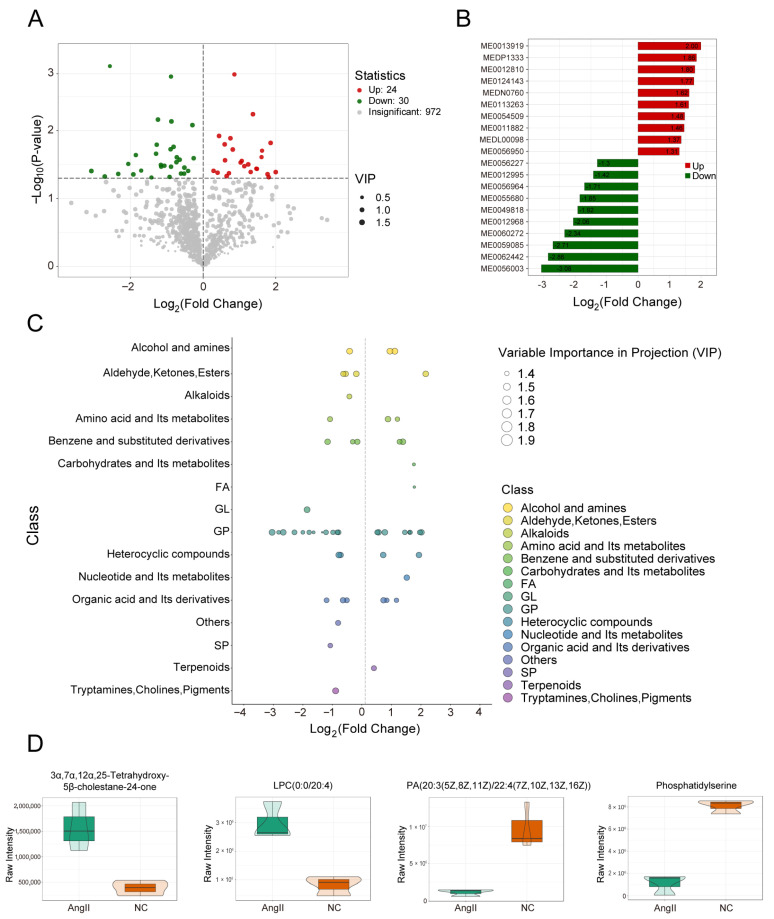
Screening of differential metabolites in VSMCs. (**A**) Metabolite volcano plot: each dot represents a metabolite, with green dots indicating downregulated differential metabolites, red dots indicating upregulated differential metabolites, and gray dots representing metabolites detected but not significantly different; the x-axis represents the Log2Fold Change(FC), which is the logarithmic value of the fold difference in relative content between the two groups of samples, where the greater the absolute value, the more significant the difference; the size of the dots represents the VIP value; (**B**) top 10 differential metabolite bar chart: the x-axis represents the Log2FC, indicating the logarithmic value of the fold difference, and the y-axis lists the differential metabolites; red bars indicate an increase in metabolite content, and green bars indicate a decrease in metabolite content; (**C**) differential metabolite scatter plot: each dot represents a metabolite, with different colors representing different categories; the x-axis indicates the logarithmic value of the fold difference in relative content Log2FC between the two groups of samples, with a greater absolute value indicating a more significant difference; the size of the dots represents the VIP value; (**D**) top differential metabolite violin plots: based on the absolute value of Log2FC, the top two differentially expressed metabolites for UP and DOWN are plotted as violin plots to show the specific changes in expression levels.

In this study, differential metabolites were screened using a combination of univariate and multivariate statistical analyses, with VIP and *p*-values as the screening criteria. Metabolites with VIP > 1 and *p* < 0.05 (Student’s *t*-test) were defined as differential metabolites (Supplemental Table S4). Among the 1026 detected metabolites, 54 differential metabolites were identified as differential, including 24 upregulated and 30 downregulated metabolites (Figure 2A). Additionally, the top 10 upregulated and downregulated metabolites, ranked by log2Fold Change (FC), were visualized using bar charts to highlight metabolites with significant differential expression (Figure 2B). The corresponding index codes are detailed in the Supplementary Table S4. Furthermore, metabolites showing the largest changes in both the upregulated and downregulated groups—including 3α, 7α, 12α, 25-tetrahydroxy-5β-cholestane-24-one, LPC (0:0/20:4), phosphatidylserine, and phosphatidic acid (20:3 (5Z, 8Z, 11Z)/22:4 (7Z, 10Z, 13Z, 16Z))—were selected for violin plot analysis to illustrate their data distributions and probability densities (Figure 2D). To further compare the relative abundance of different metabolite classes between the two groups, a differential metabolite scatter plot was generated, revealing that differential metabolites were predominantly enriched in the GP category (Figure 2C).

### 3.3. Functional Pathways of Differential Metabolites in VSMCs

**Figure 3:**
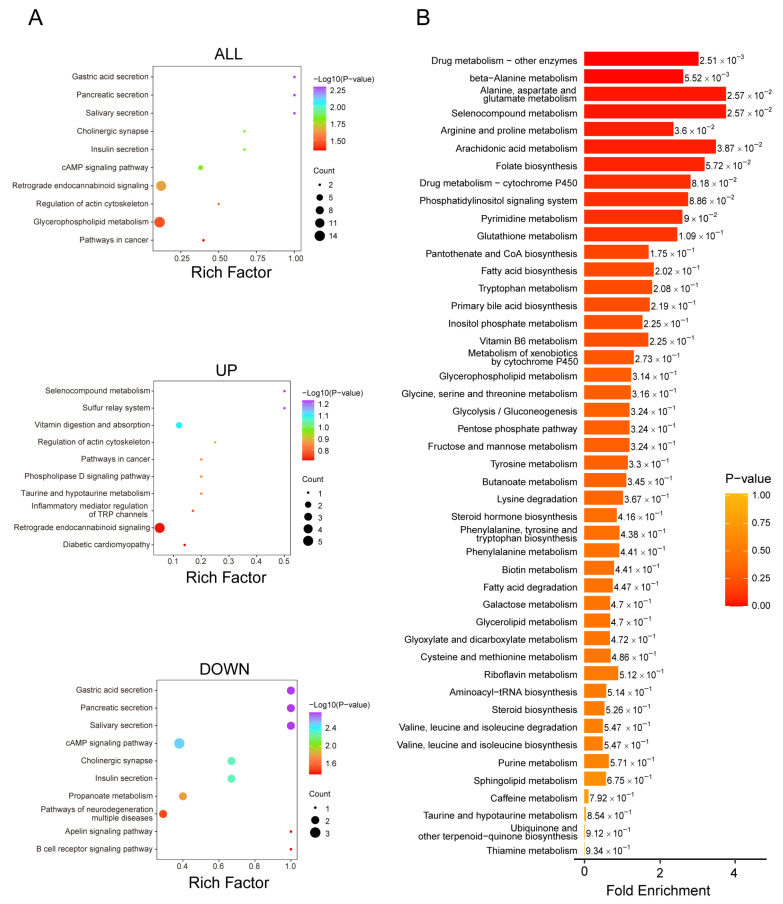
KEGG pathway and MSEA enrichment plots of differential metabolites in VSMCs. (**A**) Differential metabolite KEGG pathway enrichment bubble plot: KEGG enrichment analysis was conducted for three categories of differential metabolites, all, upregulated, and downregulated, resulting in three KEGG bubble plots. Each bubble represents a pathway: the x-axis is the Rich factor (degree of enrichment), the y-axis is the −log10 (*p*-value), the size of the bubble is proportional to the number of metabolites hit in the pathway, and the color gradient from blue to red reflects the significance from high to low; (**B**) MSEA enrichment analysis plot: based on the MSEA module of MetaboAnalyst, the differential metabolites were analyzed. The y-axis lists the names of the metabolic sets sorted by *p*-value; the x-axis is the Fold Enrichment, with a larger value indicating more significant enrichment; the color gradient corresponds to the *p*-value, with darker red indicating more reliable enrichment.

The KEGG database enables the integration of genes, expression profiles, and metabolite information into functional biological networks. MSEA does not rely on specific differential metabolite thresholds; instead, it maps metabolomics data to biologically meaningful metabolic sets and statistically identifies significantly altered pathways. In this study, KEGG pathway enrichment analysis (Figure 3A) and MSEA (Figure 3B) were performed using the screened differential metabolites, and the top 50 metabolic sets ranked by *p*-value were selected in MSEA.

KEGG analysis revealed several upregulated pathways, including selenocompound metabolism, regulation of the actin cytoskeleton, pathways in cancer, the phosphatidylinositol signaling system, and taurine and hypotaurine metabolism. These changes may reflect cellular responses to oxidative stress [32,33], regulation of cell morphology and motility [34], and enhanced cell proliferation and migration [35,36]. In contrast, downregulated pathways included gastric acid secretion, pancreatic secretion, salivary secretion, the cyclic adenosine monophosphate signaling pathway, and the cholinergic synapse, suggesting that Ang II exerts broad effects on digestive system functions and intracellular signal transduction [37]. We acknowledge that these pathway enrichments do not imply VSMCs possess secretory or neural functions; rather, they likely reflect changes in shared metabolic substrates or signaling intermediates that participate in multiple physiological contexts. MSEA demonstrated a significant enrichment of pathways such as drug metabolism—other enzymes, β-alanine metabolism, alanine, aspartate and glutamate metabolism, and selenocompound metabolism. Enrichment of the drug metabolism pathways suggests that Ang II stimulation may enhance the capacity of VSMCs to metabolize and eliminate xenobiotics or toxins. Enrichment of β-alanine metabolism may reflect alterations in energy metabolism and nitrogen balance [38]. Increased activity in alanine, aspartate, and glutamate metabolism may indicate changes in amino acid synthesis and catabolism. Furthermore, enrichment of selenocompound metabolism may be associated with enhanced antioxidant defense and cellular protective mechanisms [33].

Collectively, KEGG and MSEA analyses indicate that Ang II stimulation markedly alters metabolic activities in VSMCs, involving multiple aspects, such as drug and amino acid metabolism, antioxidant defense, cell signaling, cytoskeletal dynamics, energy metabolism, and digestive and secretory functions. These metabolic changes are consistent with the known biological effects of Ang II on vasoconstriction, cell proliferation, migration, and vascular remodeling, and provide metabolomic evidence for understanding the mechanism supporting the mechanistic role of Ang II in VSMCs.

### 3.4. Overall Metabolic Changes in Mouse Aortic Tissue Induced by Ang II

**Figure 4:**
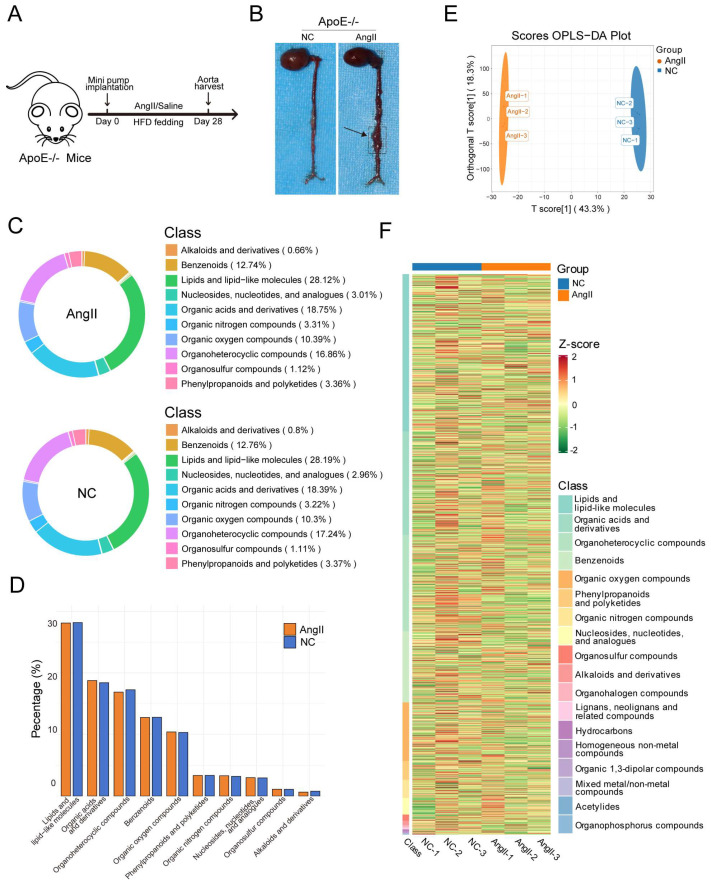
Analysis of the impact of AngII on metabolites in mouse aortic tissue. (**A**) Flowchart of mouse model establishment: ApoE−/− mice were implanted with subcutaneous osmotic minipumps, injected with either AngII or saline, and then fed a high-fat diet for 28 days to obtain aortic tissue; (**B**) appearance of mouse aorta: the aortic section of mice in the experimental group (treated with AngII) shows aneurysmal changes; (**C**) metabolite category composition pie charts: each color represents a category of metabolites, and the area of the segments indicates the proportion of each category. Two pie charts are presented, one for the NC group and one for the AngII group. Minor categories with very low abundances are omitted for visual clarity; (**D**) percentage bar graphs: these graphs display the percentages of the top ten metabolite categories in both groups, highlighting the differences between groups more clearly; (**E**) OPLS-DA score plot: the x-axis represents the predictive principal component (used to distinguish between group differences), and the y-axis represents the orthogonal principal component (used to observe differences within groups); (**F**) overall sample clustering plot: the horizontal axis represents sample names, and the vertical axis represents metabolite information. Different colors represent the standardized processing results of different relative contents (red for high content, green for low content).

After 28 days of Ang II infusion and high-fat diet feeding, the entire aorta was isolated from both the control and experimental groups (Figure 4A). Visual comparison of the specimens confirmed successful aneurysm formation in the experimental group, as evidenced by the marked dilation of the abdominal aorta (Figure 4B). The suprarenal segment of the aorta from each group was then excised for metabolomic analysis. Similarly, untargeted metabolomics identified a total of 1808 secondary metabolites across both experimental and control groups. Table 3 shows the detailed identification counts.

We analyzed the compositional proportions of metabolites and found subtle variations between the experimental and control groups (Figure 4C). As shown in the figure, both the categories and relative abundances of metabolites differ between the experimental (Ang II) and control (NC) groups. Although the proportions of most metabolite categories were comparable in both groups, subtle variations were observed in several categories, including organic acids and derivatives and nucleotides, nucleosides, and analogs. These findings suggest that Ang II treatment may selectively influence specific metabolic pathways, resulting in altered proportions of certain metabolite classes (Figure 4D). Instrument stability, detection reliability, and sample quality were verified through a series of QC analyses, all of which met the required standards (Supplementary Figure S4 and Table S5). PCA provided an initial overview of the metabolic differences between groups and the variability among samples (Supplementary Figure S2). Furthermore, OPLS-DA demonstrated a clear separation in the metabolic composition between the experimental and control groups with a satisfactory predictive reliability (R^2^Y = 1.000, Q^2^ = 0.921), further confirming the impact of Ang II treatment on metabolic profiles (Figure 4E and Figure S3). A clustering heatmap was generated using R to visualize metabolite expression patterns across all samples (Figure 4F), revealing distinct differences in multiple metabolite categories between the experimental and control groups.

### 3.5. Screening of Differential Metabolites in Aortic Tissue

**Figure 5:**
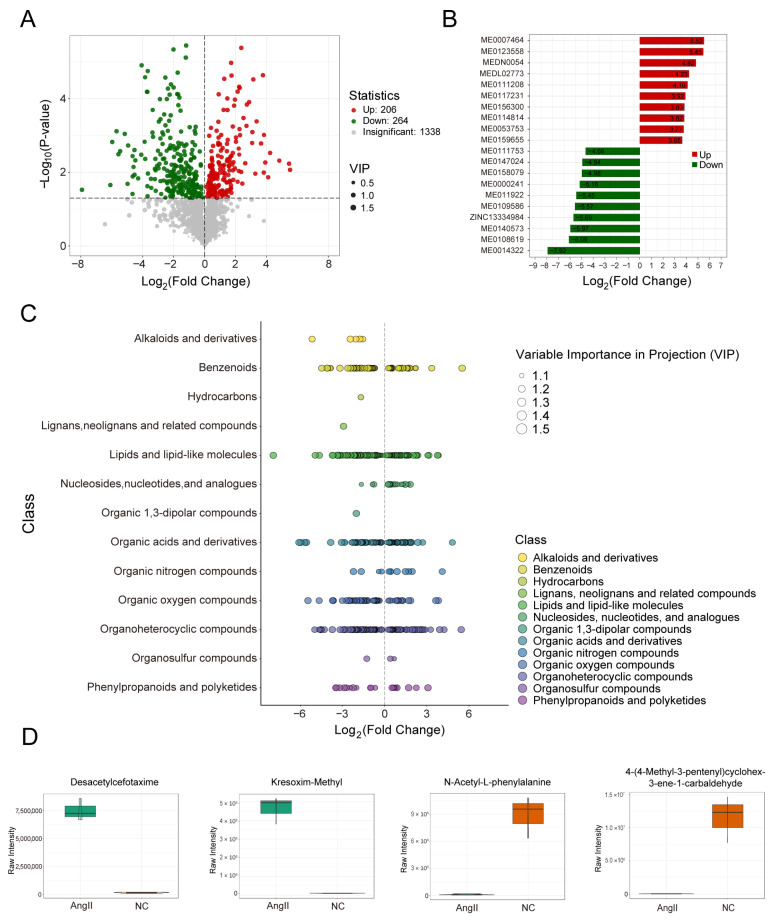
Screening of differential metabolites in aortic tissue. (**A**) Metabolite volcano plot: each dot represents a metabolite. Green dots indicate downregulated differential metabolites, red dots indicate upregulated differential metabolites, and gray dots represent metabolites with no significant difference. The x-axis is the Log2Fold Change(FC), with a larger absolute value indicating a more significant difference. The size of the dots corresponds to the VIP value; (**B**) top 10 differential metabolite bar chart: the x-axis is the Log2FC, and the y-axis lists the differential metabolites. Red bars indicate an increase in metabolite levels, and green bars indicate a decrease in metabolite levels; (**C**) differential metabolite scatter plot: each dot represents a metabolite, with different colors representing different categories of metabolites. The x-axis is the Log2FC, with a larger absolute value indicating a more significant difference. The size of the dots corresponds to the VIP value; (**D**) top differential metabolite violin plots: based on the absolute value of Log2FC, the top two differentially expressed metabolites for UP and DOWN are plotted as violin plots to illustrate the specific changes in expression levels.

Differential metabolites were identified by combining univariate and multivariate statistical analyses using consistent screening criteria, yielding 206 upregulated and 264 downregulated metabolites. These results are presented in a volcano plot (Figure 5A). Furthermore, the top 10 upregulated and downregulated metabolites, ranked by log2FC, are shown in bar charts (Figure 5B), and the corresponding index codes are provided in the Supplementary Materials. Additionally, violin plots were generated for the four metabolites exhibiting the greatest changes in both upregulated and downregulated groups, illustrating their data distributions and probability densities (Figure 5D). The corresponding index codes are detailed in Supplementary Table S6. A scatter plot was also constructed to compare the relative abundances of metabolite classes between the two sample groups, revealing that differential metabolites were predominantly enriched in benzenoids, lipids and lipid-like molecules, organic acids and derivatives, organic oxygen compounds, and organoheterocyclic compounds (Figure 5C).

### 3.6. Functional Pathways of Differential Metabolites in Aortic Tissue

**Figure 6:**
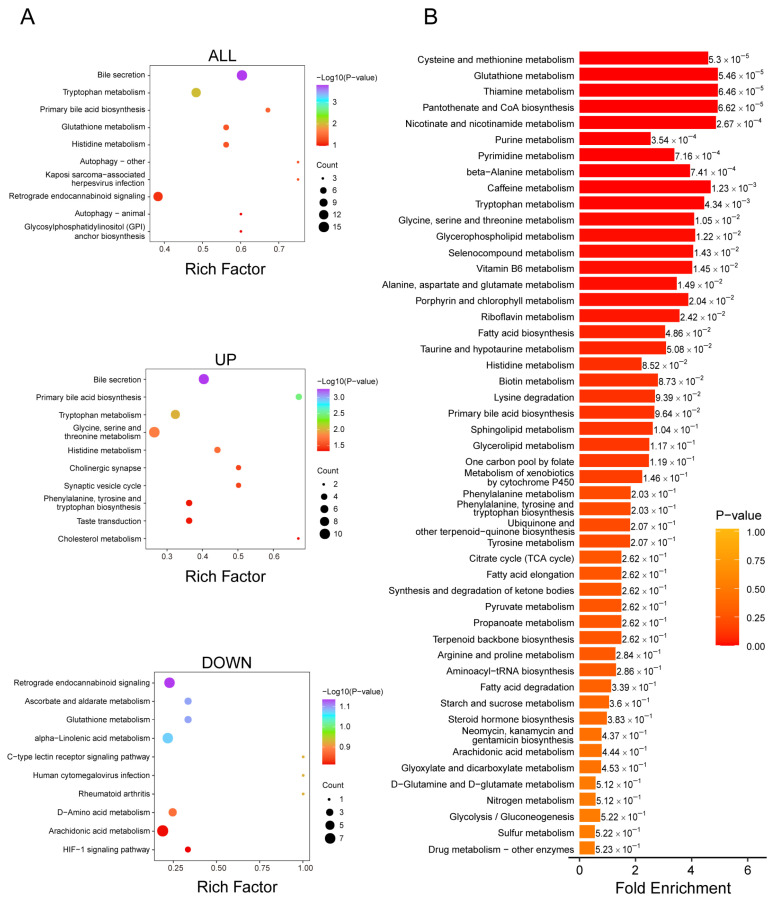
KEGG pathway and MSEA enrichment plots of differential metabolites in aortic tissue. (**A**) Differential metabolite pathway enrichment plot (KEGG): KEGG analysis was performed on all, upregulated, and downregulated differential metabolites, resulting in three bubble plots. The x-axis represents the enrichment factor, and the y-axis represents −log10 (*p*-value). The size of the bubbles indicates the number of metabolites in the pathway, and the color gradient from blue to red signifies decreasing significance; (**B**) MSEA enrichment analysis plot: differential metabolites were analyzed using the MSEA module of MetaboAnalyst. The y-axis lists the names of the metabolic sets ordered by *p*-value, and the x-axis represents the fold enrichment, with darker red indicating more significant enrichment.

We also conducted an in-depth analysis of the screened differential metabolites using two enrichment analysis approaches: KEGG pathway analysis and MSEA. KEGG enrichment analysis revealed significant changes in metabolic pathways associated with vascular inflammation and lipid metabolism (Figure 6A). Specifically, the upregulation of the “bile secretion” and “primary bile acid biosynthesis” pathways suggests the activation of lipid metabolic processes under Ang II stimulation, potentially influencing cholesterol homeostasis [39]. Meanwhile, the enrichment of the “tryptophan metabolism” pathway implies a possible involvement in vascular inflammation, as tryptophan is a precursor of serotonin and melatonin, which are involved in immune regulation and inflammatory responses [40]. Additionally, the downregulation of “glutathione metabolism” may contribute to inflammatory processes, as glutathione is a key intracellular antioxidant that helps reduce oxidative stress and cellular damage—key drivers of inflammation [41]. The downregulation of “retrograde endocannabinoid signaling” may influence the release of inflammatory mediators [42], while the downregulation of “ascorbate and aldarate metabolism” may impact antioxidant defense mechanisms [43].

MSEA further demonstrated that multiple metabolic pathways were significantly enriched under experimental conditions (Figure 6B). Pathways including “cysteine and methionine metabolism,” “glutathione metabolism,” and “thiamine metabolism” exhibited high enrichment scores, indicating their potential importance in the response to Ang II stimulation. Moreover, nucleotide metabolic pathways such as “purine metabolism” and “pyrimidine metabolism” were also significantly enriched, suggesting a modulation of nucleotide metabolism. Notably, “glutathione metabolism” was consistently enriched in both KEGG and MSEA analyses, highlighting the potential involvement of glutathione-dependent antioxidant mechanisms. Furthermore, pathways such as “tryptophan metabolism” and “bile secretion” showed significant changes in the KEGG analysis, further supporting their roles in metabolic regulation and inflammatory responses.

The integration of KEGG and MSEA results suggests enhanced metabolic regulation and altered antioxidant defense under Ang II stimulation, particularly involving glutathione- and tryptophan-related pathways. These findings provide mechanistic insights into metabolic and inflammatory alterations in mouse aortic tissue following Ang II exposure.

### 3.7. Intersection Analysis

**Figure 7:**
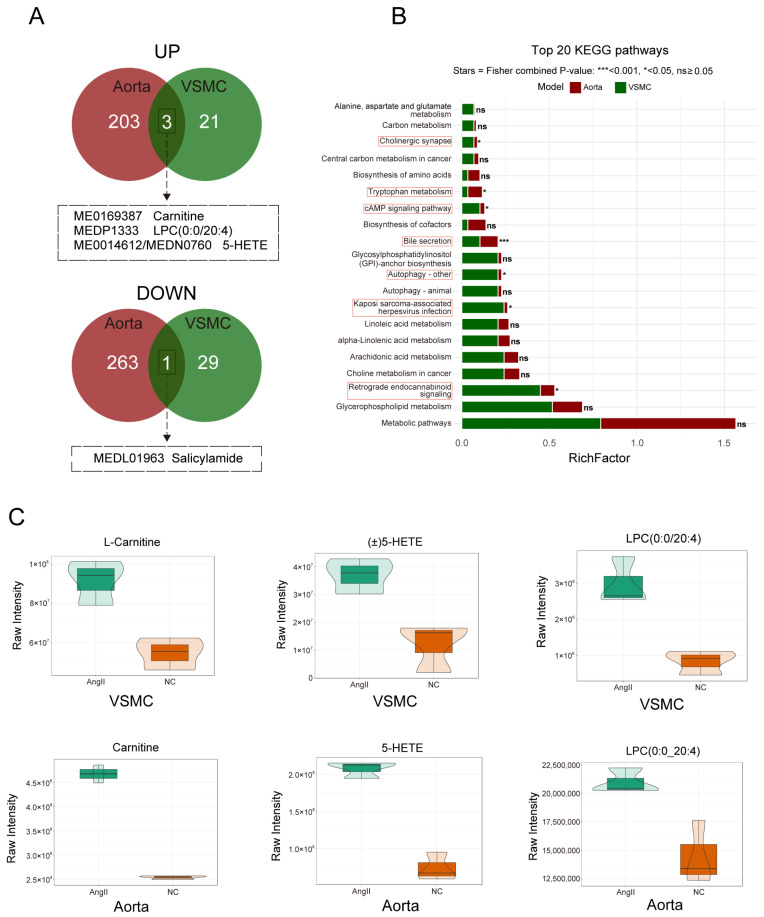
Intersection analysis of differential metabolites and their related pathways in aorta and VSMCs treated with AngII. (**A**) Intersection Venn diagram of differential metabolites: the intersection of upregulated and downregulated differential metabolites under two experimental conditions. The diagram shows three commonly upregulated metabolites (Carnitine, LPC (0:0/20:4), 5-HETE) and one commonly downregulated metabolite (Salicylamide) in both Aorta and VSMCs; (**B**) top 20 KEGG pathway bar chart: the top 20 intersecting KEGG pathways obtained from both experiments are plotted in a bar chart, with the Bile secretion pathway being the most significant; (**C**) violin plots of intersecting differential metabolites: the changes in the three intersecting differential metabolites in the Aorta and VSMC groups.

After conducting separate metabolomic analyses on the two experimental sample sets, we identified commonalities by determining the intersection of their differential metabolites. Specifically, we intersected the upregulated and downregulated metabolites from both datasets (Figure 7A). Three metabolites were consistently upregulated: carnitine, LPC (0:0/20:4), and 5-HETE. Notably, in the VSMC group, 5-HETE was detected as (±)5-HETE, resulting in a discrepancy in metabolite indexing between the two groups. Among the downregulated metabolites, only salicylamide was shared. Subsequently, the re-examination of spectral data and database annotations revealed insufficient confidence in the identification of salicylamide, suggesting potential misannotation by the metabolomics software. This compound has therefore been excluded from our biological conclusions. To visualize the magnitude of these changes, violin plots were generated for the three intersecting metabolites (Figure 7C). It should be noted that violin plots display the distribution of three shared metabolites within each model separately; data were not normalized across species or platforms. Using the KEGG and HMDB databases, we further identified disease associations for these three differential metabolites; Table 4 summarizes these findings. Similarly, we intersected the KEGG pathways enriched in both sample sets. The *p*-values were merged using Fisher’s method, and significance markers were assigned based on the combined *p*-values (Overall_*p*) (Figure 7B). Among the commonly enriched pathways, bile secretion was the most significant, with an Overall_*p* < 0.001.

## 4. Discussion

We utilized untargeted metabolomics to investigate the effects of Ang II on VSMCs and aortic tissue, generating a continuous metabolic profile spanning cellular to tissue levels. This approach enabled us to explore the association between Ang II-induced metabolic changes in VSMCs and the pathobiology of AAA. Our initial findings identified three common differentially expressed metabolites and highlighted the carnitine–bile secretion axis as a potential therapeutic target, offering novel insights into AAA pathogenesis from a metabolic perspective.

Ang II stimulation induced significant metabolic changes in VSMCs, with 54 differential metabolites identified—24 upregulated and 30 downregulated. These changes are associated with pathways including drug metabolism, amino acid metabolism, antioxidant defense, and lipid metabolism. In aortic tissue, 470 differential metabolites were detected, with significant alterations in pathways related to bile acid metabolism, tryptophan metabolism, and glutathione metabolism. Together, these findings indicate that Ang II triggers extensive metabolic remodeling in VSMCs, potentially contributing to AAA development and progression.

Among the identified metabolites, three—carnitine, LPC (0:0/20:4), and 5-HETE—were found in both VSMCs and aortic tissue. These metabolites have been implicated in various pathological processes, including AAA. Carnitine, for instance, can be metabolized by specific gut microbiota into trimethylamine-N-oxide [44], a known promoter of AAA that contributes to hypertension and aortic dissection [45,46,47]. In our study, carnitine was upregulated in both VSMCs and aortic tissue under Ang II stimulation, confirming its involvement in AAA pathogenesis. LPC and 5-HETE are key mediators of inflammation [48]. Research indicates that LPC induces cyclooxygenase-2 and promotes pro-inflammatory responses, while 5-HETE, an arachidonic acid metabolite, acts as a central inflammatory mediator. The upregulation of both LPC and 5-HETE in VSMCs and aortic tissue suggests a role in inflammation-driven AAA progression.

KEGG enrichment analysis revealed that carnitine participates in the bile secretion pathway in both VSMC and aortic metabolomics sequencing. However, we must acknowledge that vascular smooth muscle cells do not possess physiological bile secretory function. Currently, the two main targets for bile acid action are the farnesoid X receptor (FXR) and the G protein-coupled bile acid receptor 1 (GPBAR1, also known as TGR5) [49]. Given that FXR is predominantly expressed in the vascular smooth muscle layer, whereas GPBAR1 is primarily localized to the vascular endothelium, we have shifted our discussion focus to the “carnitine–FXR signaling axis” as a potential mechanism underlying Ang II-induced AAA pathophysiology. Existing research indicates that carnitine facilitates fatty acid β-oxidation [50], while bile acids function as signaling molecules to regulate inflammatory responses by activating FXR [51]. Therefore, we hypothesize that, under Ang II stimulation, carnitine in VSMCs may influence bile acid synthesis and secretion by modulating fatty acid β-oxidation. This metabolic shift could impair FXR-mediated vascular protection, promote inflammatory responses, facilitate the synthetic phenotype transition of VSMCs, and exacerbate aortic inflammation, collectively contributing to AAA progression. Additionally, our experimental results showed that the elevation of carnitine levels occurred without significant changes in the expression of its regulatory genes (Supplementary Figure S5), suggesting that carnitine accumulation in our models may be governed by post-transcriptional or post-translational mechanisms rather than simple transcriptional activation. Such regulatory complexity may involve multiple layers of metabolic control, including enzyme kinetics, substrate availability, and systemic metabolic influences. These findings highlight the complexity of metabolic regulation in AAA, and future studies are warranted to elucidate the underlying mechanisms through dedicated mechanistic investigations. The depletion of glutathione metabolism in aortic tissue represents a particularly noteworthy finding. Glutathione serves as the primary intracellular antioxidant, and its metabolic downregulation suggests a compromised redox buffering capacity within the aneurysmal wall [52]. This impairment may accelerate disease progression by amplifying oxidative stress-induced vascular damage, promoting inflammatory cascades (particularly through TNF and NOD-like receptor signaling pathways), and sensitizing vascular smooth muscle cells to degenerative stimuli [53]. We recognize that glutathione metabolism likely interacts with lipid and bile acid signaling pathways in complex ways that warrant future investigation.

Despite our findings, this study has several limitations. First, our research primarily relied on in vitro VSMC and animal models, without validation in clinical samples. Future studies should assess the clinical relevance of these metabolic pathways and biomarkers in human AAA patients. Moreover, the differential metabolites identified in this study should be regarded as exploratory findings requiring validation in larger cohorts with appropriate multiple testing correction. Second, although we identified several potential metabolic pathways via metabolomics analysis, the specific molecular mechanisms of these pathways remain unclear. In particular, the role of the carnitine–FXR signaling axis in AAA requires further investigation using genetic knockout, pharmacological interventions, and mechanistic studies. Third, the VSMC and aortic tissue models yielded substantially different numbers of differential metabolites (54 versus 470), reflecting their distinct biological scales and complexities. These differences arise from inherent methodological distinctions: the VSMC model captures acute, cell-autonomous metabolic responses, whereas the murine model encompasses chronic, multi-cellular pathological processes. Future studies should therefore incorporate additional experimental approaches, including primary VSMCs and alternative aneurysm induction models, to corroborate these findings across diverse methodological contexts. Finally, while our results suggest the carnitine–FXR signaling axis as a potential therapeutic target, its application prospects in AAA treatment require cautious evaluation. Future research should further explore its safety and efficacy in preclinical models to provide a scientific basis for the development of new therapeutic strategies based on metabolic regulation.

## 5. Conclusions

In summary, this study employed untargeted metabolomics to elucidate the association between Ang II-induced metabolic changes in VSMCs and the pathobiology of AAA, identifying the carnitine–FXR signaling axis as a potential therapeutic target. These findings provide new insights into the metabolic mechanisms underlying AAA pathogenesis and establish a foundation for the future development of metabolically informed diagnostic biomarkers and therapeutic strategies. Further research is needed to validate the clinical relevance of these metabolic pathways in human AAA and to explore their underlying mechanisms and translational potential.

## Figures and Tables

**Table 1:** Primer Information.

Primer Names	Primer Sequences (5′–3′)	Fragment Lengths (bp)
AGTR1a-F AGTR1a-R	ACAACTGCCTGAACCCTCTG TTGTAAGAGGTGCCCTGGAAG	144
MMP2-F Mmp2-R	AGTATGGGAACGCTGATGGC TTGTAAGAGGTGCCCTGGAAG	238
MMP9-F Mmp9-R	GCAAACCCTGCGTATTTCCATT GCGATAACCATCCGAGCGAC	83
VCAM1-F VCAM1-R	CCACTAAATGGGAAGGTGAAGAC GGTAAACATCAGGAGCCAAACAC	235
β-ACTIN-F β-ACTIN-R	TGCTATGTTGCCCTAGACTTCG GTTGGCATAGAGGTCTTTACGG	240

**Table 2:** VSMC metabolite identification count table.

Ion Mode	ALL	T3_Positive	T3_Negative
Number of metabolites	1178	659	519
Number of secondary identified metabolites	1026	544	482

**Table 3:** Mouse aortic tissue metabolite identification count table.

Ion Mode	ALL	T3_Positive	T3_Negative
Number of metabolites	2003	999	1004
Number of secondary identified metabolites	1808	863	965

**Table 4:** Intersection of differential metabolites associated with diseases table.

Compound Name	Hmdb Diseases
Carnitine	Colorectal cancer | Early preeclampsia | Pregnancy | Late-onset preeclampsia | L-2-Hydroxyglutaric aciduria | 3-Hydroxy-3-Methylglutaryl-CoA Synthase Deficiency | 3-Hydroxyacyl-CoA dehydrogenase deficiency | 3-Hydroxy-3-methylglutaryl-CoA lyase deficiency | 2,4-dienoyl-CoA reductase deficiency | Myopathic carnitine deficiency | Carnitine palmitoyltransferase I deficiency | Carnitine transporter defect | primary systemic carnitine deficiency | Methylmalonic aciduria mitochondrial encephelopathy Leigh-like | Mitochondrial trifunctional protein deficiency | Long-chain Fatty Acids, Defect in Transport of | Oculocerebrorenal syndrome | Perillyl alcohol administration for cancer treatment | Periodontal disease | Pancreatic cancer | Attachment loss | Missing teeth | Periodontal Probing Depth | Tooth Decay | Diabetes mellitus type 2 | Lung Cancer | Eosinophilic esophagitis | Propionic acidemia | Obesity
LPC (0:0/20:4)	-
5-HETE	Asthma | Rheumatoid arthritis | Rhinitis

## Data Availability

The data supporting this study are provided in the article and Supplementary Materials. Differential metabolite lists are available in Supplementary Tables S4 and S6. Raw data are available from the corresponding author upon reasonable request.

## Supplementary Materials

The following supporting information can be downloaded at: https://www.mdpi.com/article/10.3390/biomedicines14030623/s1, Figure S1: Quality Control (QC) assessment in both positive-ion and negative-ion modes for Ang II-treated VSMCs; Figure S2: Principal Component Analysis (PCA) score plot of all samples from the two models; Figure S3: OPLS-DA permutation test validation for metabolomic models; Figure S4: QC assessment in both positive-ion and negative-ion modes for Ang II-treated mouse aortas; Figure S5: Validation of carnitine metabolism in VSMC and aortic tissue models; Table S1: LC–MS/MS gradient conditions; Table S2: Mass spectrometry parameters; Table S3: QC data of VSMCs; Table S4: Differential metabolites in VSMCs; Table S5: QC data of mouse aortic tissue; Table S6: Differential metabolites in mouse aortic tissue.

## Abbreviations

The following abbreviations are used in this manuscript:

AAA	Abdominal aortic aneurysm
Ang II	Angiotensin II
ApoE−/−	Apolipoprotein E knockout
AT1R	Angiotensin II type 1 receptor
CV	Coefficient of variation
DMEM	Dulbecco’s Modified Eagle Medium
FXR	Farnesoid X receptor
GP	Glycerophospholipids
HMDB	Human Metabolome Database
IACUC	Institutional Animal Care and Use Committee
KEGG	Kyoto Encyclopedia of Genes and Genomes
KNN	K-nearest neighbor
LC–MS/MS	Liquid chromatography–tandem mass spectrometry
LPC	Lysophosphatidylcholine
MMP2	Matrix metalloproteinase 2
MMP9	Matrix metalloproteinase 9
MSEA	Metabolite set enrichment analysis
NC	Normal control
OPLS-DA	Orthogonal partial least squares discriminant analysis
PCA	Principal component analysis
QC	Quality control
qPCR	Quantitative polymerase chain reaction
RT	Retention time
SVR	Support vector regression
TIC	Total ion current
TMAO	Trimethylamine-N-oxide
UHPLC	Ultra-high-performance liquid chromatography
VIP	Variable importance in projection
VSMCs	Vascular smooth muscle cells
5-HETE	5-Hydroxyeicosatetraenoic acid
