# Supplementary material for: The Metabolite Differences in Vascular Smooth Muscle Cells of Abdominal Aortic Aneurysm Revealed by Untargeted Metabolomics
Source: Biomedicines. 2026 Mar 11;14(3):623. doi: 10.3390/biomedicines14030623 (PMC13023614; doi:10.3390/biomedicines14030623)
Supplement: Supplementary file 1 [file biomedicines-14-00623-s001.zip › Supplementary Figures.pdf]

Supplementary Materials

The Metabolite Differences in Vascular Smooth Muscle Cells of Abdominal Aortic Aneurysm Revealed by Untargeted Metabolomics

Yuqi Yi [1,2,+], Ke Hu [1,2,+], Yuxuan Li [3,+] , Jie Li[1,2,*] and Hongping Deng [1,2,*]

[1] Department of Vascular Surgery, Renmin Hospital of Wuhan University, Wuhan 430060, China; 2024283020136@whu.edu.cn (Y.Y.); rm004312@whu.edu.cn(K.H.)

[2] Aortic Abdominal Aneurysm (AAA) Translational Medicine Research Center of Hubei Province, Wuhan 430060, China

[3] Department of Pancreatic Surgery, Union Hospital, Tongji Medical College, Huazhong University of Science and Technology, Wuhan 430000, China; d202482182@hust.edu.cn

* Correspondence: rm003171@whu.edu.cn (J.L.); hpdeng@whu.edu.cn (H.D.)

\+ These authors contributed equally to this work.

Supplementary Figures

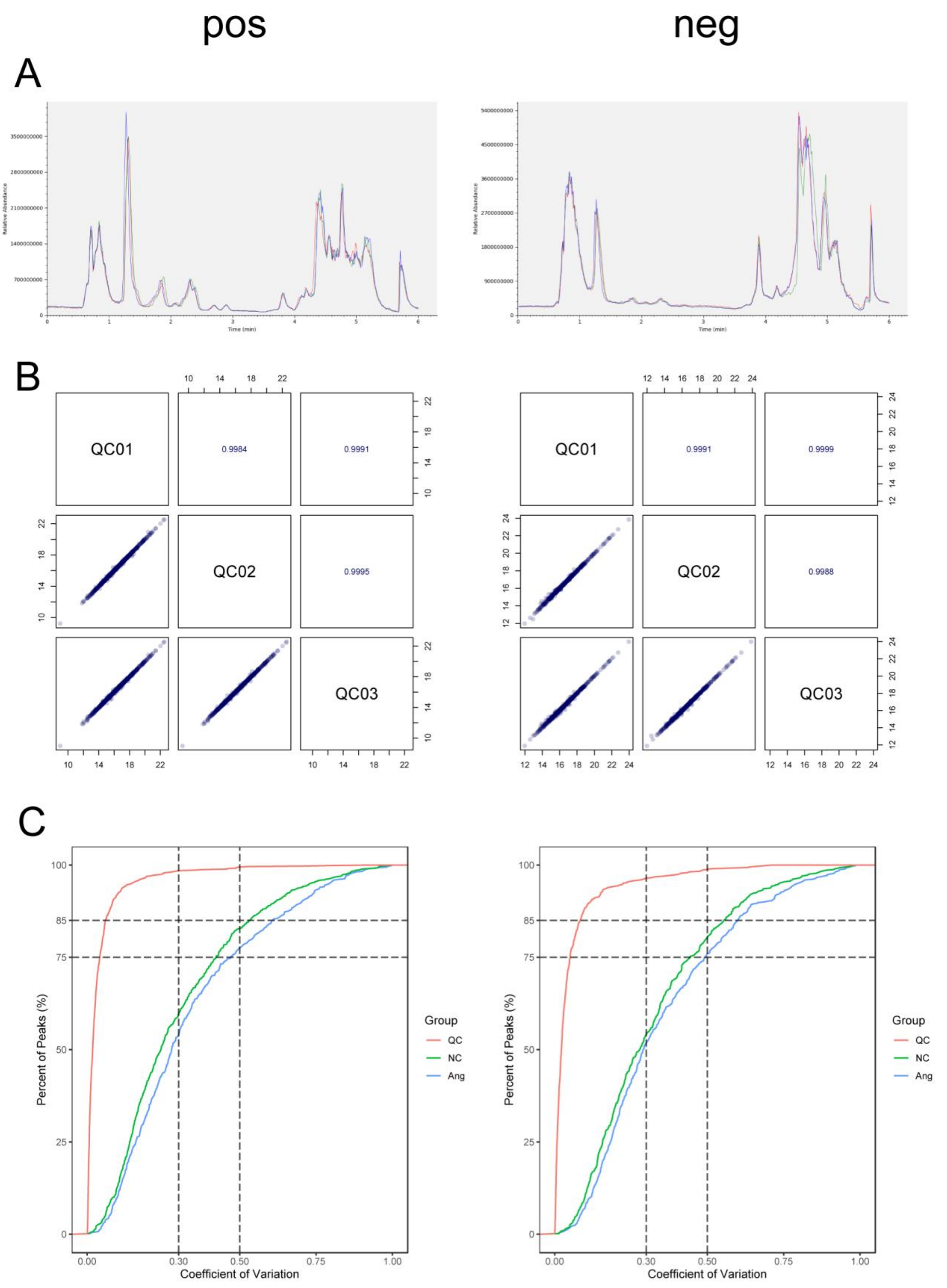

Figure S1. Quality Control (QC) assessment in both positive-ion and negative-ion modes for Ang II–treated VSMCs. (**A**) QC-sample total-ion-current overlay: The near-perfect alignment of retention time and peak intensity across injections confirms high signal stability. (**B**) QC sample correlation analysis: Pearson correlation of QC samples: |r| approaching 1 indicates high system stability and data

quality. (**C**) CoefficientofVariation (CV) -distribution plot: x-axis, CV; y-axis, fraction of features below that CV. QC (quality-control) samples shown in red; a higher proportion of low-CV features in QC indicates greater analytical stability.

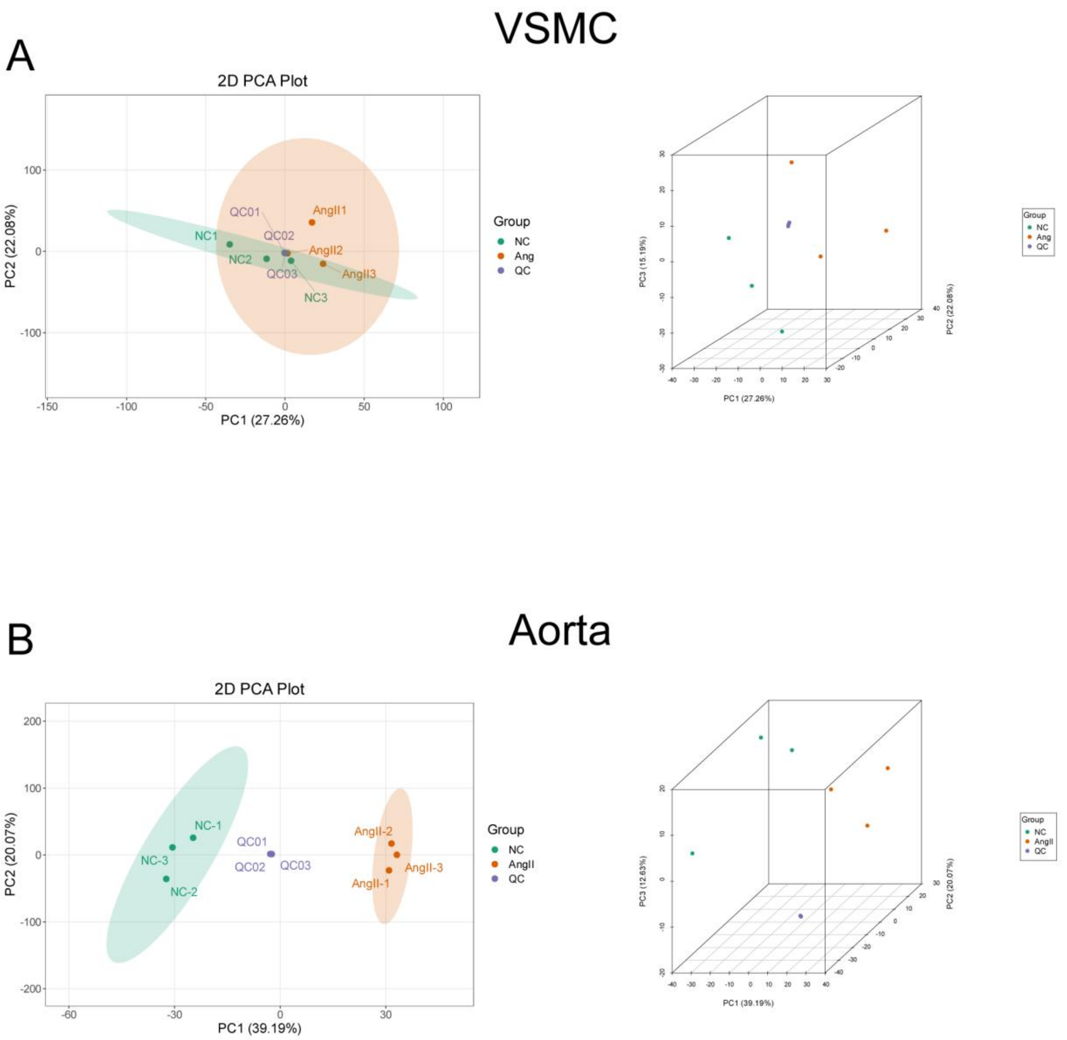

Figure S2. Principal Component Analysis (PCA) score plot of all samples from the two models. (**A**) PCA score plot (2D and 3D) of LC-MS profiles from Ang II–treated VSMC groups: PC1, PC2 and PC3 denote the first, second and third principal components; percentages indicate variance explained. Each dot represents one sample; colours denote experimental groups. (**B**) PCA score plot (2D and 3D) of LC-MS profiles from Ang II–treated mouse aortas groups: PC1, PC2 and PC3 denote the first, second and third principal components; percentages indicate variance explained. Each dot represents one sample; colours denote experimental groups.

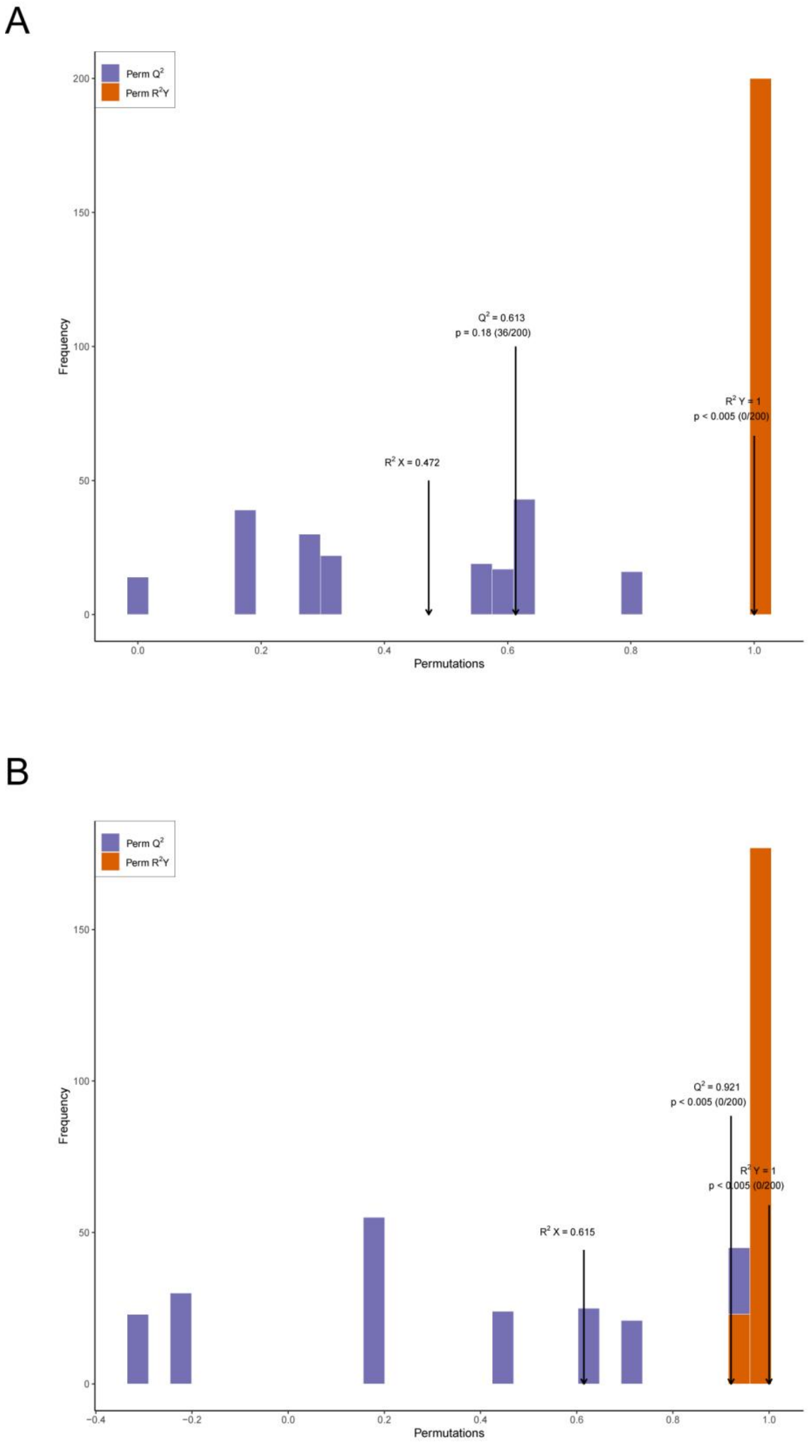

Figure S3. OPLS-DA permutation test validation for metabolomic models. Permutation tests ($n = 200$) were performed to validate the robustness of OPLS-DA models. (A) Rat VSMC model: $Q^2 = 0.613$, $R^2Y = 1.000$, $p < 0.005$. (B) Mouse aortic tissue model: $Q^2 = 0.921$, $R^2Y = 1.000$, $p < 0.005$. Perm Q^2 (blue bars) and Perm R^2Y (orange bars) represent the distributions of Q^2 and R^2Y values obtained from permuted datasets. The actual Q^2 and R^2Y values (indicated by arrows) substantially exceed the permuted distributions, confirming that the observed group separation is not attributable to random chance.

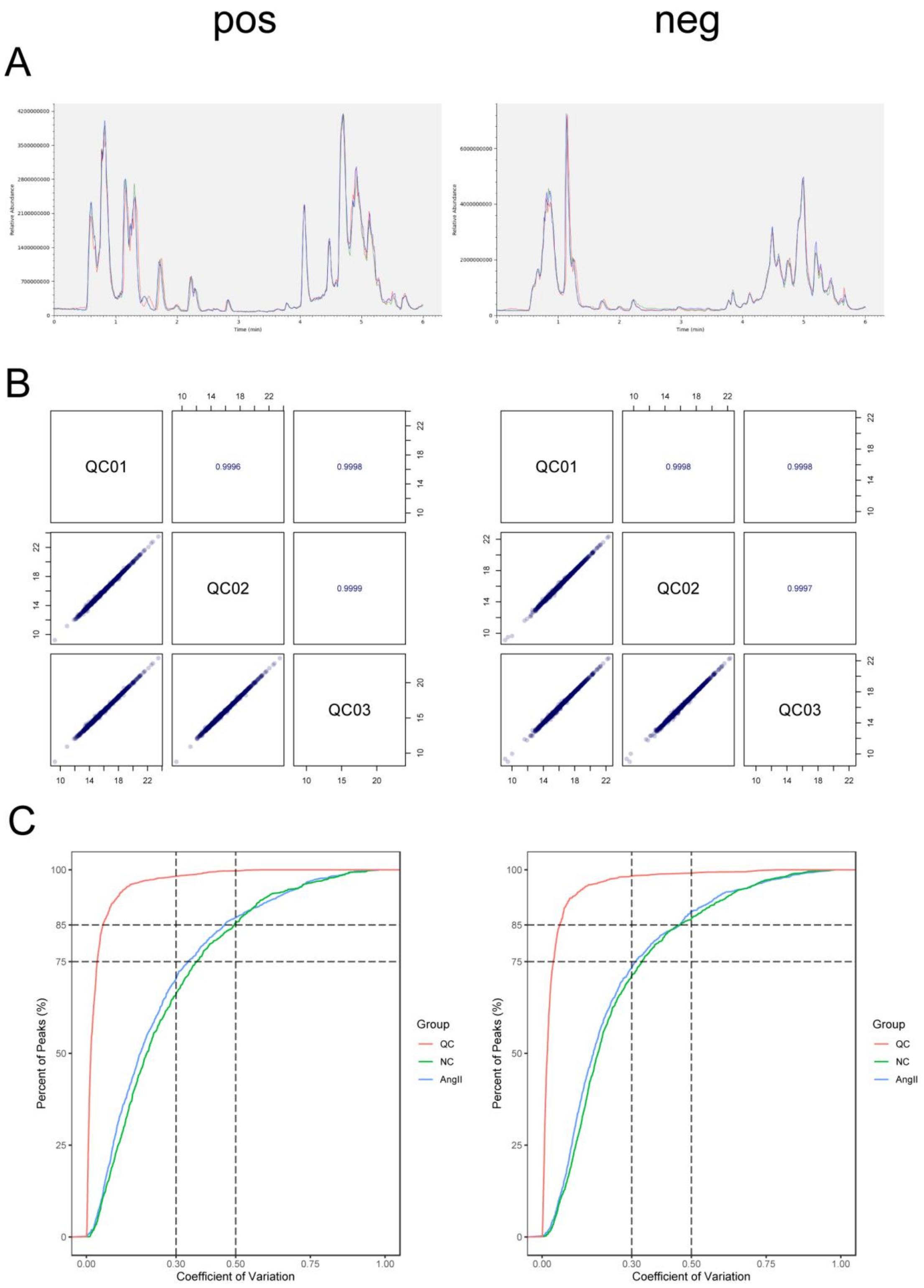

Figure S4. Quality Control (QC) assessment in both positive-ion and negative-ion modes for Ang II–treated mouse aortas. (**A**) QC-sample total-ion-current overlay: The near-perfect alignment of retention time and peak intensity across injections confirms high signal stability. (**B**) QC sample correlation analysis: Pearson correlation of QC samples: |r| approaching 1 indicates high system stability and data quality. (**C**) CoefficientofVariation (CV) -distribution plot: x-axis, CV; y-axis, fraction

of features below that CV. QC (quality-control) samples shown in red; a higher proportion of low-CV features in QC indicates greater analytical stability.

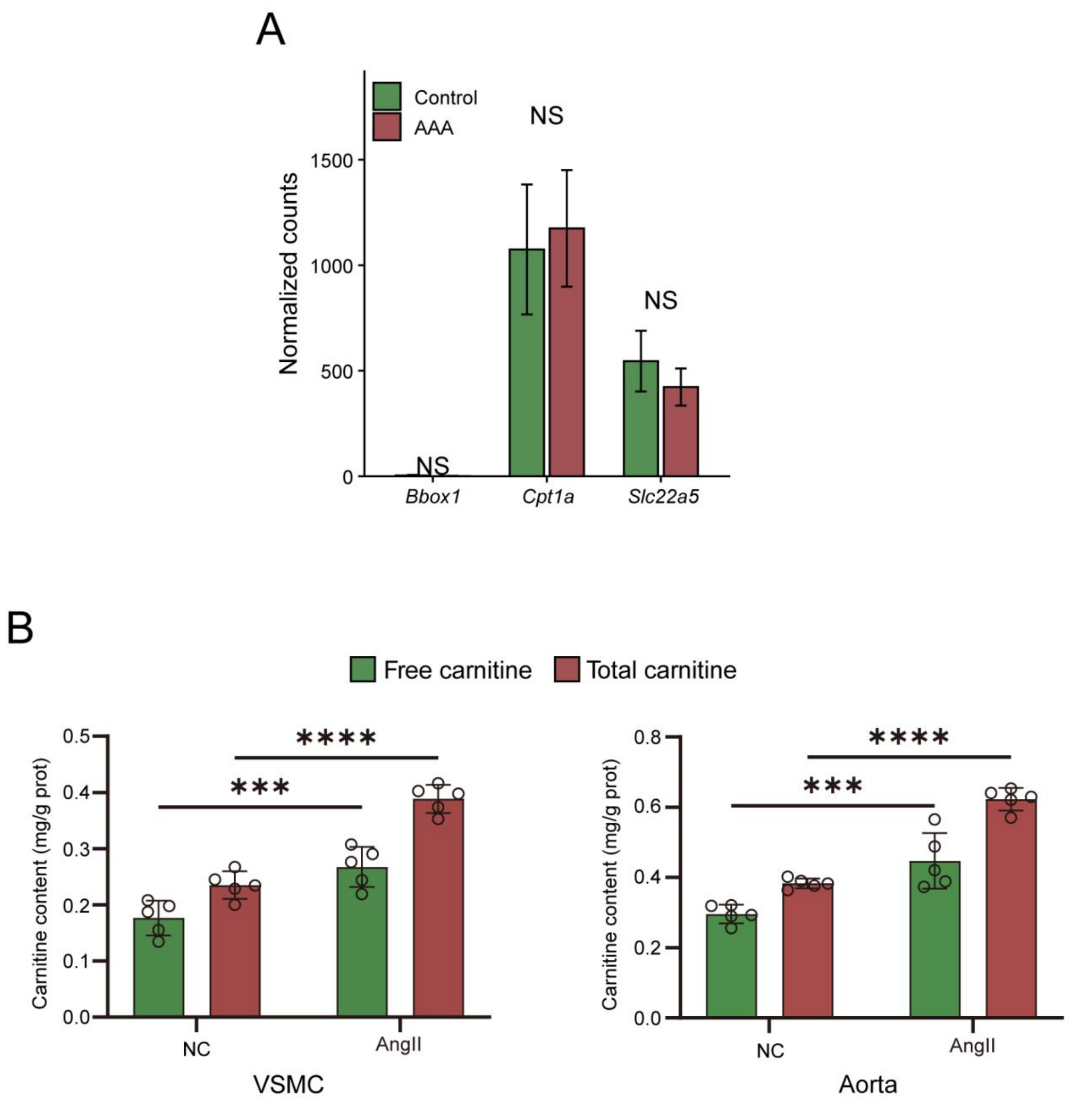

Figure S5. Validation of carnitine metabolism in VSMC and aortic tissue models. (A) mRNA expression levels of carnitine-related genes (*Bbox1*, *Cpt1a*, and *Slc22a5*) in control and AAA aortic tissues. Data are presented as normalized counts with no significant differences (NS) observed between groups. (B) Quantification of free carnitine and total carnitine levels in VSMCs (left panel) and aortic tissue (right panel) using a commercial assay kit. Free carnitine and total carnitine levels were significantly elevated in both models following Ang II stimulation compared to normal control (NC). Data are expressed as mg/g protein. Statistical significance was determined by Student's t-test: ***P < 0.001, ****P < 0.0001; NS, not significant.
